# Apelin-36-[L28A] and Apelin-36-[L28C(30kDa-PEG)] peptides that improve diet induced obesity are G protein biased ligands at the apelin receptor

**DOI:** 10.1016/j.peptides.2019.170139

**Published:** 2019-11

**Authors:** Duuamene Nyimanu, Rhoda E. Kuc, Thomas L. Williams, Maria Bednarek, Philip Ambery, Lutz Jermutus, Janet J. Maguire, Anthony P. Davenport

**Affiliations:** a Experimental Medicine and Immunotherapeutics, University of Cambridge, Level 6, Centre for Clinical Investigation, Box 110, Addenbrooke’s Hospital, Cambridge, CB2 0QQ, UK; b Research and Early Development, Cardiovascular, Renal and Metabolism (CVRM), BioPharmaceuticals R&D, AstraZeneca, Cambridge, UK; c Late-stage Development, Cardiovascular, Renal and Metabolism (CVRM), BioPharmaceuticals R&D, AstraZeneca, Gothenburg, Sweden

**Keywords:** Apelin, Apelin receptor, Biased ligands, Metabolic disease, Cardiovascular disease, Human heart, Receptor affinity, GPCR

## Abstract

**Background:**

Apelin signalling pathways have important cardiovascular and metabolic functions. Recently, apelin-36-[L28A] and apelin-36-[L28C(30kDa-PEG)], were reported to function independent of the apelin receptor in vivo to produce beneficial metabolic effects without modulating blood pressure. We aimed to show that these peptides bound to the apelin receptor and to further characterise their pharmacology in vitro at the human apelin receptor.

**Methods:**

[Pyr^1^]apelin-13 saturation binding experiments and competition binding experiments were performed in rat and human heart homogenates using [^125^I]apelin-13 (0.1 nM), and/or increasing concentrations of apelin-36, apelin-36-[L28A] and apelin-36-[L28C(30kDa-PEG)] (50pM-100μM). Apelin-36 and its analogues apelin-36-[F36A], apelin-36-[L28A], apelin-36-[L28C(30kDa-PEG)], apelin-36-[A28 A13] and [40kDa-PEG]-apelin-36 were tested in forskolin-induced cAMP inhibition and β–arrestin assays in CHO-K1 cells heterologously expressing the human apelin receptor. Bias signaling was quantified using the operational model for bias.

**Results:**

In both species, [Pyr^1^]apelin-13 had comparable subnanomolar affinity and the apelin receptor density was similar. Apelin-36, apelin-36-[L28A] and apelin-36-[L28C(30kDa-PEG)] competed for binding of [^125^I]apelin-13 with nanomolar affinities. Apelin-36-[L28A] and apelin-36-[L28C(30kDa-PEG)] inhibited forskolin-induced cAMP release, with nanomolar potencies but they were less potent compared to apelin-36 at recruiting β-arrestin. Bias analysis suggested that these peptides were G protein biased. Additionally, [40kDa-PEG]-apelin-36 and apelin-36-[F36A] retained nanomolar potencies in both cAMP and β-arrestin assays whilst apelin-36-[A13 A28] exhibited a similar profile to apelin-36-[L28C(30kDa-PEG)] in the β–arrestin assay but was more potent in the cAMP assay.

**Conclusions:**

Apelin-36-[L28A] and apelin-36-[L28C(30kDa-PEG)] are G protein biased ligands of the apelin receptor, suggesting that the apelin receptor is an important therapeutic target in metabolic diseases.

## Highlights

• Apelin-36-[L28A] and apelin-36-[L28C(30kDa-PEG)] were initially reported to induce beneficial metabolic effects independent of the apelin receptor in vivo.
• We have shown that these peptides activate the apelin receptor, competing for binding with nanomolar affinities in rat and human cardiac tissues.
• Apelin-36-[L28A] and apelin-36-[L28C(30kDa-PEG)] were more potent at inhibiting cAMP release compared to β-arrestin activation.
• Our study suggests that apelin-36-[L28A] and apelin-36-[L28C(30kDa-PEG)] are G protein biased ligands of the apelin receptor.

## 1 Introduction

Apelin is the endogenous ligand of the previously orphaned G protein-coupled apelin receptor [1]. Apelin-36 was first identified in 1998 from bovine stomach extracts and is suggested to be the cleavage product of a 77-amino acid pre(pro)apelin [2]. Based on paired dibasic cleavage sites, the existence of shorter fragments of the peptide was predicted and to date the most characterised isoforms are apelin-36, apelin-17 and apelin-13 [3]. [Pyr^1^]apelin-13 was identified as the most predominant isoform in rat plasma and hypothalamus [4]; human heart [5] and human plasma [6]. Lower levels of apelin-17 were also identified in rat hypothalamus and plasma [4].

In mammals, apelin and its receptor are widely expressed in tissues including the heart, brain, lungs and kidney [[7], [8], [9]] and a number of physiological actions have been proposed for this peptide system [10,11]. Importantly, cardiovascular and metabolic roles are the most characterised. In the cardiovascular system, apelin is a vasodilator [12,13], strong positive inotrope [5,14,15], and promotes angiogenesis [16]. Apelin increases insulin sensitivity by promoting increased glucose uptake in skeletal muscle [11], and apelin knockout mice develop hyperinsulinemia and insulin resistance without any apparent differences in body weight [17,18]. Additionally, in both normal and diet-induced obese mice apelin decreased adiposity, serum insulin and triglycerides, without modulating food intake [19]. These beneficial roles of apelin in regulating metabolism and energy expenditure have ignited interest in developing apelin analogues as a therapeutic strategy for improved management of metabolic diseases.

In order to further characterise the physiological effects of apelin in vivo, Galon-Tilleman et al. [20] generated a series of apelin-36 variants with the aim of understanding the structure-activity relationship of the peptide and extending its plasma stability. This series included a leucine-28 to alanine substitution (apelin-36-[L28A]) to test whether this site can tolerate further modifications, and subsequently a leucine-28 to cysteine substitution that allowed PEGylation of the resulting cysteine-28 (apelin-36-[L28C(30kDa-PEG)]), an established strategy to dramatically increase the plasma half-life of peptides by reducing proteolysis and renal excretion [21]. They also tested a second site within the peptide, the C-terminal phenylalanine to alanine substitution (apelin-36-[F36A]) and its corresponding N-terminal PEGylated analogue, [40kDa-PEG]-apelin-36. Finally they generated apelin-36-[A13 A28] which involves alanine substitutions at positions 13 and 28 [20]. The authors reported that these peptides potently prevented diet-induced obesity by improving glucose tolerance, and decreasing blood glucose levels, cholesterol and LDL. However, surprisingly, they did not observe an effect on blood pressure in vivo which would be expected for apelin receptor agonists [20]. Apelin-36-[L28A] and apelin-36-[L28C(30kDa-PEG)] also showed marked impaired activation of the apelin receptor in a β-arrestin assay compared to apelin-36. The authors concluded that the metabolic action was independent of the apelin receptor and suggested that there could be another as yet unidentified receptor for apelin. However, the ability of these compounds to inhibit radiolabelled apelin binding was not determined. These beneficial metabolic actions represent a potential new target. It is crucial to determine the mechanism of action and whether or not they are mediated via the apelin receptor. Therefore, our aim was to obtain direct evidence for binding of these analogues to the apelin receptor in competition-binding experiments in rat and human heart. Secondly, to compare relative potencies of the analogues compared with apelin-36 in G protein dependent (cAMP) and G protein independent (β-arrestin) in vitro assays.

## 2 Methods

### 2.1 Reagents and chemicals

[Pyr^1^]apelin-13 was purchased from Severn Biotech (Kidderminster, UK) and [Glp^65^, Nle^75^, Tyr^77^][^125^I]apelin-13 (subsequently referred to as [^125^I]apelin-13) was synthesised by Perkin Elmer (MA, USA). Apelin-36 and its analogues apelin-36-[F36A]), apelin-36-[L28A], apelin-36-[L28C(30kDa-PEG)], [40kDa-PEG]-apelin-36 and apelin-36-[A13 A28] were obtained from AstraZeneca (UK) Plc. AGTRL1 β-Arrestin (Lot No.: 93-0250E2) and cAMP (Lot No.: 95-0147E2) assays were purchased from DiscoverX (Fremont, USA). Protein assay reagents where obtained from Bio-Rad (USA, cat #500-0116); protease inhibitor cocktail (Cat No.: P8340) that inhibits a broad range of proteases including serine, cysteine and acid proteases, and aminopeptidases, was purchased from Sigma. All other reagents were obtained from Sigma.

### 2.2 Human and rat heart homogenate preparation

Samples of human left ventricle were obtained with informed consent and ethical approval (05/Q104/142) via the Papworth Hospital Research Tissue Bank (08/H0304/56) from the explanted hearts of five dilated cardiomyopathy patients (4 males and 1 female, aged 38–55 years) undergoing transplantation. Studies conformed to the principles outlined in the declaration of Helsinki. All animal research was regulated under the Animals (Scientific Procedures) Act 1986 Amendment Regulations 2012 following ethical review by the University of Cambridge Animal Welfare and Ethical Review Body (AWERB). Adult female and male Sprague Dawley rats (n = 14; aged 7–10 weeks) were sacrificed using overdose of CO_2_ and cervical dislocation before heart tissue was removed and frozen on dry ice. Hearts were pooled and 2 g tissue transferred into a centrifuge tube (50 ml) containing 20 ml homogenisation buffer (50 mM Tris, 5 mM MgCl_2_, 5 mM EDTA, 1 mM EGTA at pH 7.4 with protease inhibitor cocktail) on ice. The tissue was homogenised on ice using a polytron probe with 2–3 × 20 s bursts and centrifuged at 3000 rpm for 2 min at 4 °C. The supernatant was further centrifuged at 18,500 rpm for 30 min at 4 °C. The resulting pellet was resuspended in 5 ml homogenisation buffer containing protease inhibitor cocktail and centrifuged at 18,500 rpm for 30 min at 4 °C twice. Final pellets were then resuspended in 10 ml HEPES buffer (50 mM HEPES, pH 7.4) and protein concentration determined. Samples were stored at −70 °C until required.

### 2.3 Protein concentration

Protein concentration was determined using Bio-Rad DC Protein Assay (Bio-Rad, Cat. #500-0116) according to manufacturer’s protocol. Briefly, 3.75 mg/ml BSA was prepared in solubilisation buffer (0.5 M NaOH, 1% SDS) and diluted to a final BSA standard concentration range of 0.1875–1.5 mg/ml in the same buffer. The homogenate sample (100 μl) was then solubilised by mixing with 100 μl solubilisation buffer, before incubation for 30 min at 80 °C. Samples were subsequently centrifuged at 11000xg for 5 min at room temperature before adding 5 μl of the supernatant, blank or BSA standard to empty wells of a 96-well plate. A 25 μl aliquot of Reagent A’ comprising 2.5 ml reagent A and 50 μl reagent S per plate was added to each well before adding 200 μl/well of reagent B and incubating for 5 min at room temperature. Absorbance at 750 nm was read using an EL800 Universal Microplate Reader (Bio-Tek Instruments).

### 2.4 Saturation binding experiments

Prior to carrying out the competition binding assays to determine affinities of the apelin-36 analogues, it was necessary to find the affinity of the radiolabelled apelin-13 compound by saturation analysis in rat and human heart. Radioligand binding assays were carried out according to Read et al. [22]. Rat and human heart homogenates were diluted to 4.5 mg/ml (1.5 mg/ml final concentration) in assay buffer (Tris 50 mM, MgCl_2_ 5 nM, pH 7.4). [^125^I]apelin-13 was diluted in assay buffer to achieve a final concentration range of 2 nM-3.9 pM. Protein samples (100 μl) were incubated with increasing concentrations of [^125^I]apelin-13 (100 μl), in the absence (total binding) or presence of 2 μM [Pyr^1^]apelin-13 (to define non-specific binding as this concentration will compete for >98% of total binding obtained with 2 nM [^125^I]apelin-13), for 90 min at 4 °C, until equilibrium was reached. Samples were then centrifuged at 20,000xg for 10 min at 4 °C to terminate the reaction. The resulting pellets were washed with 500 μl ice-cold wash buffer (Tris−HCl buffer; 50 mM Tris, pH 7.4) before a second centrifugation at the same settings. Pellets were counted on a Cobra II Auto-Gamma radiation counter (Packard) to obtain radioactivity in disintegrations per minute (DPM). These data were analysed using the KELL program to determine the concentration of [^125^I]apelin-13 that occupies 50% of the apelin receptors (K_D_, the equilibrium dissociation constant) and apelin receptor density (B_max_) [23]. All tubes and pipette tips were treated with Sigmacote (Sigma-Aldrich, Gillingham, UK) to reduce non-specific binding of the radiolabel to plastic consumables.

### 2.5 Radioligand competition binding assay

Competition binding experiments were performed using pooled human homogenised left ventricle (HLV, 1.5 mg/ml) and pooled rat heart homogenates (1.5 mg/ml) as previously described [24,25]. Homogenates were incubated for 90 min with [^125^I]apelin-13 (0.1 nM) in assay buffer (Tris 50 mM, MgCl_2_ 5 nM, pH 7.4), in the presence of increasing concentrations of apelin-36, apelin-36-[L28A] or apelin-36-[L28C(30kDa-PEG)] (50 pM-100 μM). Binding in the presence of 2 μM [Pyr^1^]apelin-13 was considered non-specific. The samples were centrifuged at 20,000xg for 10 min, at 4 °C before pellets were washed with Tris−HCl buffer (50 mM, pH 7.4) on ice to remove any unbound ligands. The samples were then re-centrifuged to collect protein pellets before bound radioactivity in these pellets were counted using Packard Cobra II Auto Gamma Counter (Packard). Data were analysed with Graphpad Prism 7 (Graphpad Software, Inc. La Jolla, CA), using the One-site Fit K_i_ equation. The K_D_ values (human heart, 0.076 nM; rat heart, 0.28 nM) for the radiolabel were obtained from the saturation binding assays and subsequently used in the GraphPad Prism analysis to determine the K_i_ (equilibrium dissociation constant obtained from a competition binding experiment).

### 2.6 Inhibition of cAMP accumulation assay

Cell-based cAMP assay were performed as previously described [22,24]. Briefly, Chinese Hamster Ovary cells (CHO-K1) cells artificially expressing the human apelin receptor were seeded in Cell Plating media into 96-well plates and incubated for 24 h at 37 °C in 5% CO_2_. The media was replaced with cAMP Antibody Reagent in Cell Assay Buffer before adding 15 μM forskolin in the presence of human [Pyr^1^]apelin-13, human apelin-36, apelin-36-[F36A], apelin-36-[L28A], apelin-36-[L28C(30kDa-PEG)], apelin-36-[A13 A28] and [40kDa-PEG]-apelin-36 (1 pM-0.3 μM), followed by 30 min incubation at 37 °C in 5% CO_2_. Cells were subsequently incubated with cAMP Detection Reagent comprising cAMP Lysis Buffer, Substrate Reagent 1, Substrate Reagent 2 and cAMP Solution D for 1 h at room temperature followed by 3 h incubation with cAMP Reagent A at room temperature and chemiluminescence reading (LumiLITE™ Microplate Reader, DiscoverX). Responses were fitted to a 4-parameter logistic equation in GraphPad Prism 7 (La Jolla, CA, USA) and values of potency, pD_2_ (-log_10_ EC_50_, where EC_50_ is the concentration producing half maximal response) determined. Data were expressed as percentage inhibition of forskolin-stimulated cAMP production.

### 2.7 β-Arrestin recruitment assay

Experiments were performed using a β-arrestin GPCR Assay (AGTRL1) (DiscoverX, Lot No.: 16L0902) according to methods previously described [22,24]. CHO-K1 cells artificially expressing the human apelin receptor were seeded in Cell Plating media into 96-well plates and incubated for 48 h at 37 °C in 5% CO_2_. Serial dilutions of human [Pyr^1^]apelin-13, human apelin-36, apelin-36-[F36A], apelin-36-[L28A], apelin-36-[L28C(30kDa-PEG)], apelin-36-[A13 A28] and [40kDa-PEG]-apelin-36 were made in Cell Plating media from a 1 mM stock solution (in water). A 5 μl aliquot of apelin-36, apelin-36-[F36A], apelin-36-[L28A], apelin-36-[L28C(30kDa-PEG)], apelin-36-[A13 A28] and [40kDa-PEG]-apelin-36 was used to construct concentration-response curves in duplicate, and plates were incubated for 90 min at 37 °C, in 5% CO_2_ before Detection Reagent (27.5 μl/well) was added and further incubated for 2 h at room temperature in the dark. Negative controls were defined with equal volumes of drug solvent (water). The resulting chemiluminescent signal was measured using a LumiLITE™ Microplate Reader (DiscoverX, Fremont, CA). Responses, measured in relative light units, were fitted with a 4-parameter logistic equation in GraphPad Prism 7 (La Jolla, CA, USA) and values of pD_2_ and maximum response (E_Max_) were calculated for each compound. The data were subsequently normalised to the maximum response to [Pyr^1^]apelin-13 used as reference agonist (positive control) in the assay.

### 2.8 Statistical analysis

Data were expressed as mean ± SEM. Experiments were performed in triplicates or duplicates and n-values were expressed as number of independent experiments. Potencies of analogues in cell-based assays were compared to native apelin-36 using a one-way ANOVA with Dunnett’s posthoc test in GraphPad Prism 7. A *P* value < 0.05 was considered statistically significant. Binding affinities in both species were compared using Student’s *t*-test in Graphpad Prism as described above. Bias calculations were performed as previously described using the operational model for bias [26], to obtain values for relative effectiveness of the apelin-36 synthetic analogues compared to apelin-36 within each cell-based assay. Bias factors were calculated to compare the relative activities of the analogues between the different pathways – β-arrestin (G protein independent) and cAMP (G protein dependent).

## 3 Results

### 3.1 Characterisation of [Pyr^1^]apelin-13 binding in human and rat heart

**Fig. 1:**
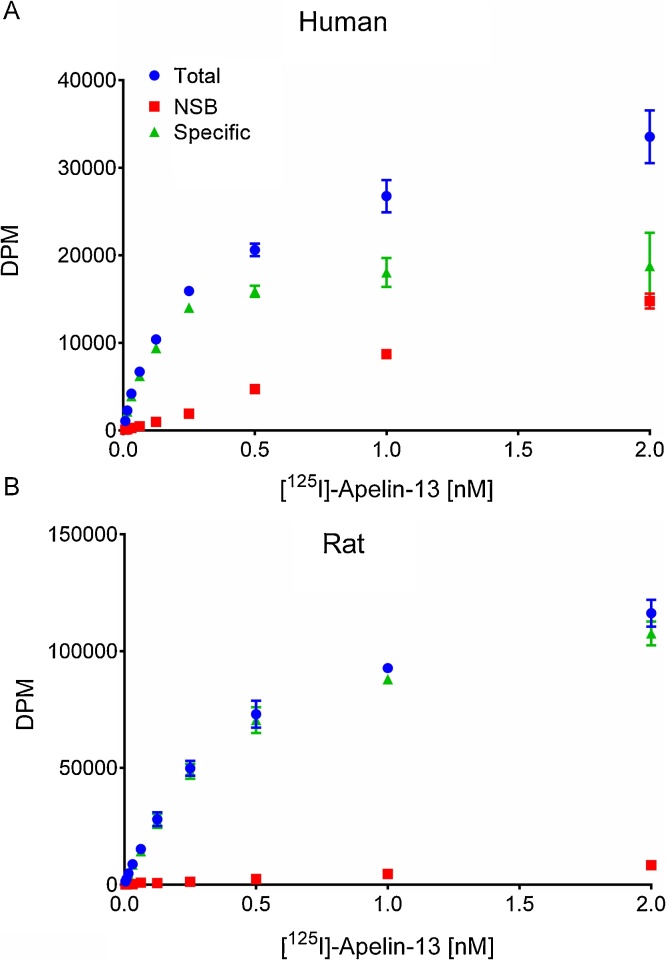
**Characterisation of binding of [^125^I]apelin-13 to rat and human cardiac apelin receptors.** [^125^I]-apelin-13 saturation binding in human (A) and rat (B) homogenates (n = 3 Replicates per concentration, data are mean ± SEM). NSB is non-specific binding defined in the presence of 2 μM [Pyr^1^]apelin-13.

We observed that in both species [Pyr^1^]apelin-13 bound with comparable single (Hill slopes were close to 1; human 0.95 ± 0.11, rat 0.95 ± 0.03) subnanomolar affinities (human K_D_, 0.08 ± 0.01 nM; rat K_D_, 0.28 ± 0.02 nM). The apelin receptor density in both species was also similar (human, 13.82 ± 1.79 fmol/mg; rat, 83.4 ± 1.85 fmol/mg) (Fig.1)

### 3.2 Apelin-36, apelin-36-[L28A] and apelin-36-[L28C(30kDa-PEG)] compete for binding to apelin receptor in rat and human heart

**Fig. 2:**
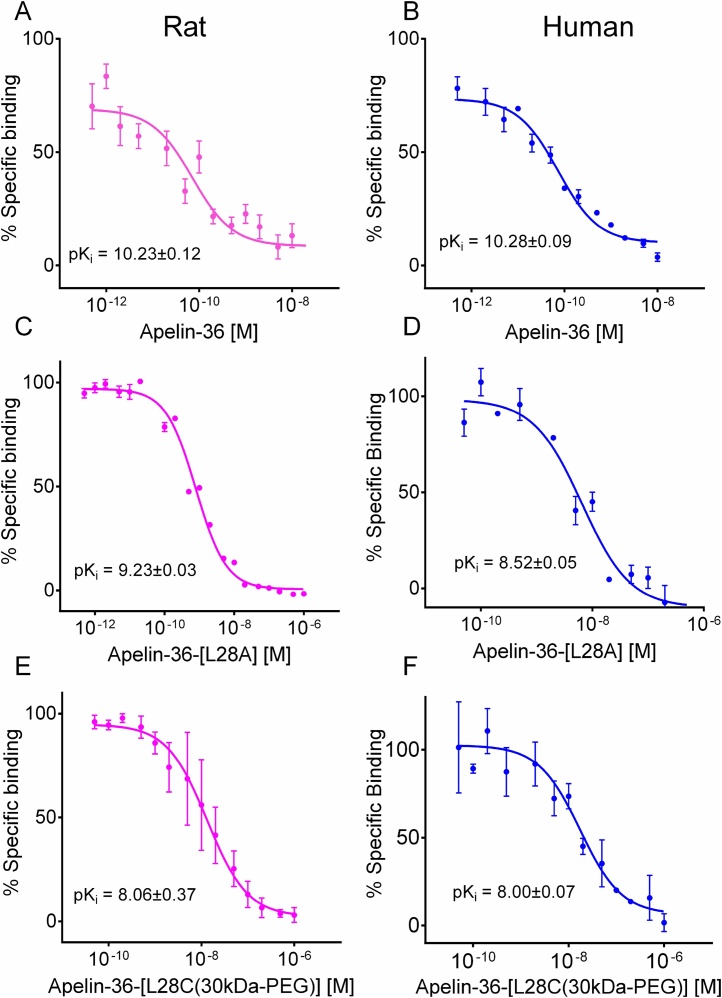
**Relative affinity of apelin-36 analogues in rat and human heart.** Apelin-36 and its analogues, apelin-36-[L28A], apelin-36-[L28C(30kDa-PEG)] bind to the apelin receptor in rat and human heart. Apelin-36 (A) Apelin-36-[L28A] (C), apelin-36-[L28C(30kDa-PEG)] (E) binding in rat heart homogenates. Apelin-36 (B) Apelin-36-[L28A] (D) and apelin-36-[L28C(30kDa-PEG)] (F) binding in human heart homogenates. n = 3 Replicates per concentration, data are mean ± SEM). pK_i_ = -log10 EC_50_ of equilibrium dissociation constant determined in a competition binding experiment (K_i_).

In rat hearts apelin-36 (pK_i_, 10.23 ± 0.12) and apelin-36-[L28A] (pK_i_ 9.51 ± 0.18) bound the receptor with subnanomolar affinities, and apelin-36-[L28C(30kDa-PEG)] with low nanomolar affinities (pK_i_, 8.06 ± 0.37) (Fig. 2A,C,E). Similarly, apelin-36 bound the human apelin receptor with subnanomolar affinity (pK_i_: apelin-36 10.28 ± 0.09) but its analogues apelin-36-[L28A] and apelin-36-[L28C(30kDa-PEG)] competed for apelin receptor binding in human heart with low nanomolar affinities (apelin-36-[L28A], 8.52 ± 0.05; apelin-36-[L28C(30kDa-PEG)], 8.00 ± 0.05), (Fig. 2B,D,F). Whereas the PEGylated apelin-36 affinity was not different for human and rat receptors, apelin-36-[L28A] had a significant 10-fold higher affinity for the rat receptor (*p* < 0.05).

### 3.3 Activity of apelin-36 analogues in cAMP assay

**Fig. 3:**
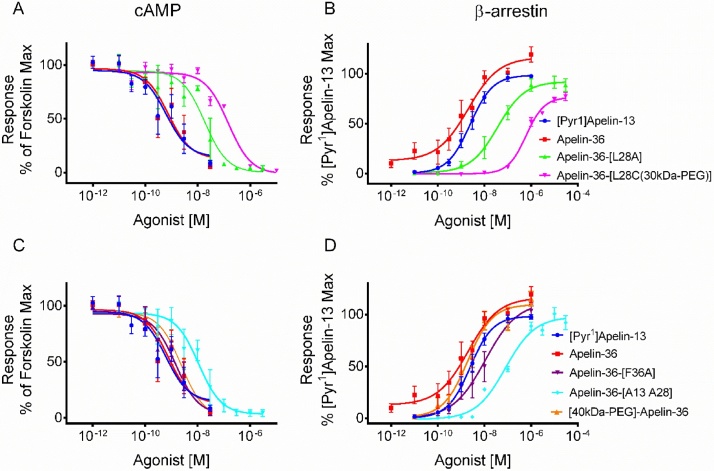
**Relative potency of apelin-36 analogues in cell-based assays**. The apelin isoforms, [Pyr^1^]apelin-13 and apelin-36 were tested in cell based β-arrestin and cAMP assays. Data are mean ± SEM of 3–4 independent experiments. pD_2_ (negative log_10_ of the concentration producing 50% of maximum response) from each peptide was compared to apelin-36 using one-way ANOVA. *P* <  0.05 was considered statistically significant.

The full sequence of the human peptides and their respective mutations are shown in Table 1. In the cAMP assay, the rank order of potencies was apelin-36 > apelin-36-[L28A] ≥ apelin-36-[L28C(30kDa-PEG)] (pD_2_: 9.04 ± 0.45 > 7.88 ± 0.24 ≥ 7.02 ± 0.09, respectively). Both analogues were less potent than apelin-36 but this was significant only for apelin-36-[L28C(30kDa-PEG)] (*p* < 0.001), (Fig. 3, Table 2). [40kDa-PEG]-apelin-36 had similar potency to the endogenous apelin-36 in the cAMP assay (pD_2_ 8.69 ± 0.22) (Fig. 3C; Table 2). Similarly, pD_2_ values for apelin-36-[F36A] and apelin-36-[A13 A28] were comparable to apelin-36 in this assay (pD_2_ 8.90 ± 0.21 and 8.12 ± 0.34, respectively).

**Table 1:** Sequences of apelin peptides showing amino acid residues affected by modifications in apelin-36 [20].

Peptides (Human)	Sequences
Apelin-13	QRPRLSHKGPMPF (full agonist)
Apelin-36	LVQPR GSRNG PGPWQ GGRRK FRRQR PRLSH KGPMP F (full agonist)
Apelin-36 (L28A)	LVQPR GSRNG PGPWQ GGRRK FRRQR PR**A**SH KGPMP F (100 fold less active at apelin receptor but full metabolic activity)
Apelin-36-[L28C(30kDa-PEG)]	LVQPR GSRNG PGPWQ GGRRK FRRQR PR **Cys(30kDa-PEG)** SH KGPMPF
Apelin-36 (F36A)	LVQPR GSRNG PGPWQ GGRRK FRRQR PRLSH KGPMP **A**
Apelin-36 (A13 A28)	LVQPR GSRNG PG**A**WQ GGRRK FRRQR PR**A**SH KGPMP F
[40kDa-PEG]-Apelin-36	**(40kDa-PEG)** LVQPR GSRNG PGPWQ GGRRK FRRQR PRLSH KGPMP F

The modifications are shown in bold. Comments refer to findings reported by Galon-Tilleman et al., [20].

**Table 2:** Summary of the relative potency of apelin-36 analogues.

Compounds	β-Arrestin Assay			cAMP Assay		
	EC_50_ (nM)	pD_2_±SEM	Fold change	EC_50_ (nM)	pD_2_±SEM	Fold change
[Pyr^1^]apelin-13	2.75	8.56 ± 0.17	**4**	0.64	9.19 ± 0.35	**0.7**
Apelin-36	0.68	9.17 ± 0.34	**1**	0.92	9.04 ± 0.45	**1**
Apelin-36-[L28A]	37.2	7.43 ± 0.07****	**55**	13.05	7.88 ± 0.24	**14**
Apelin-36-[L28C (30kDa-PEG)]	900	6.05 ± 0.06****	**1324**	95.32	7.02 ± 0.09***	**104**
Apelin-36-[F36A]	8.18	8.09 ± 0.25**	**12**	1.26	8.90 ± 0.21	**1.4**
Apelin-36-[A13 A28]	150	6.82 ± 0.14****	**221**	7.63	8.12 ± 0.34	**8**
[40kDa-PEG]-Apelin-36	1.95	8.71 ± 0.15	**3**	2.03	8.69 ± 0.22	**2**

Data are mean ± SEM n = 3–4 independent experiments. pD_2_ (negative log_10_ of the concentration producing 50% of maximum response). *Significantly different from Apelin-36 compared using ANOVA with Dunnett’s posthoc test and significance set at *p < 0.05* (** *p* <  0.01, *** *p* < 0.001, **** *p* < 0.0001).

### 3.4 Activity of the apelin-36 analogues in β-arrestin recruitment assays

In the β-arrestin assays, the lower potency obtained with apelin-36-[L28A] (pD_2_ 7.43 ± 0.07) and apelin-36-[L28C(30kDa-PEG)] (pD_2_ 6.05 ± 0.06) compared to apelin-36 (pD_2_ 9.17 ± 0.34) was more apparent than in the cAMP assay, with both analogues being significantly less potent than apelin-36 (*p* < 0.01; Fig. 3B, Table 2). Similarly, when compared to apelin-36 (pD_2_ 9.17 ± 0.34) responses to apelin-36-[F36A] (pD_2_ 8.09 ± 0.25, *p* < 0.01) and apelin-36-[A13 A28] (pD_2_ 6.82 ± 0.14, *p* < 0.0001) were significantly decreased (approximately 12- and 221-fold respectively) in this assay (Fig. 3D, Table 2). [40kDa-PEG]-apelin-36 had similar potency to the endogenous apelin-36 in the β-arrestin assay (pD_2_; 8.71 ± 0.15 vs 9.17 ± 0.34) (Fig. 3D, Table 2).

### 3.5 The apelin-36 analogues show biased signalling at the apelin receptor

**Fig. 4:**
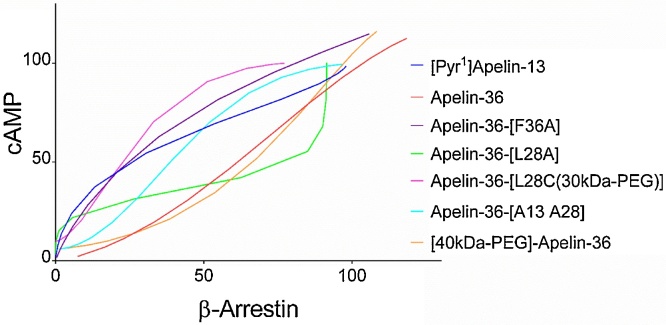
**Bias plot for apelin-36 and analogues in cAMP and β-arrestin assays.** Curves show the corresponding responses in each assay to equivalent concentrations of apelin-36 and analogues in CHO-K1 cells expressing the apelin receptor. Deviation in the shape of the curves indicates ligand bias at the receptor level. Responses in the cAMP assay were expressed as % inhibition of the forskolin response and in the β-arrestin assay as % of the maximal response to [Pyr^1^]apelin-13.

Apelin-36 and [40kDa-PEG]-apelin-36 each exhibited similar potency in the β-arrestin compared to cAMP assays although the PEGylated apelin-36 demonstrated a small drop in potency compared to the native peptide (Table 2). However, apelin-36[L28A], apelin-36-[L28C(30kDa-PEG)], apelin-36[F13A] and apelin-36-[A13 A28] were approximately 3-, 10-, 12 and 20-fold more potent in the cAMP assay compared to the β-arrestin assay respectively (Fig. 3A,B; Table 2). The degree of functional selectivity (bias) of these apelin-36 analogues for the two pathways, G protein dependent (cAMP assay) and G protein-independent (β-arrestin assay), was calculated as previously described [26], using apelin-36 as the reference ligand for this analysis. The relative effectiveness (RE), transduction ratio (logR) and bias factors are shown in Table 3, Table 4 with apelin-36[L28C-(30kDa-PEG)] showing the greatest degree of preference for the G protein pathway. These data are confirmed in the bias plot (Fig. 4) [27] that further indicates that these analogues are G protein biased at the level of the receptor in a system independent manner.

**Table 3:** Values of ΔlogR and relative effectiveness (RE) for apelin-36 analogues compared to apelin-36 in cAMP and β-arrestin assays.

Agonists	cAMP Assay		β-Arrestin Assay	
	ΔlogR	RE	ΔlogR	RE
[Pyr^1^]apelin-13	0.10 ± 0.35	1.26	−0.82 ± 0.13	0.15
Apelin-36	0.00 ± 0.43	1.00	0.00 ± 0.28	1.00
Apelin-36-[L28A]	−1.06 ± 0.35	0.09	−2.18 ± 0.17	0.01
Apelin-36-[L28C-30kDa-PEG]	−1.95 ± 0.31	0.01	−3.71 ± 0.12	0.0002
Apelin-36-[F36A]	0.25 ± 0.33	1.78	−1.19 ± 0.23	0.06
Apelin-36-[A13 A28]	−1.06 ± 0.27	0.09	−2.28 ± 0.15	0.01
[40kDa-PEG]-Apelin-36	0.08 ± 0.40	1.20	−0.59 ± 0.16	0.26

ΔlogR is the Log_10_(τ/K_A_) where τ is a measure of agonist efficacy and K_A_ is a measure of functional affinity [26]; for each assay n = 3–4 independent experiments.

**Table 4:** ΔΔlogR and bias factor for apelin-36 analogues compared to apelin-36 in cAMP and β-arrestin assays.

Agonists	cAMP vs β-arrestin Assay	
	ΔΔlogR ± SEM	Bias factor
[Pyr^1^]apelin-13	0.92 ± 0.37	8
Apelin-36	0.00 ± 0.51	1
Apelin-36-[L28A]	1.12 ± 0.39	13
Apelin-36-[L28C-30kDa-PEG]	1.76 ± 0.33**	58
Apelin-36-[F36A]	1.44 ± 0.40 *	28
Apelin-36-[A13 A28]	1.23 ± 0.31 *	17
[40kDa-PEG]-Apelin-36	0.67 ± 0.43	5

ΔΔlogR is the difference between ΔlogR values in both pathways (cAMP or G protein and β-arrestin pathways). *Significantly different from Apelin-36 compared using Student’s *t*-test with significance set at *p < 0.05* (* *p* <  0.05; ** *p* < 0.01).

## 4 Discussion

We report on the pharmacodynamic characteristics of apelin-36 analogues that were designed to have longer plasma stability, some of which were proposed to exert apelin receptor independent effects [20]. We have now demonstrated that apelin-36-[L28A] and apelin-36-[L28C(30kDa-PEG)] do bind to the apelin receptor in human and rat heart where they competed for binding with [^125^I]apelin-13 with nanomolar affinities. These data therefore imply that the reported beneficial metabolic mechanism of action for these analogues is likely through the apelin receptor. Compared with the sub-nanomolar affinity of apelin-36 in heart from both species, the apelin-36 L28A mutation resulted in an order of magnitude reduction in affinity and this was further reduced in the PEGylated analogue; this may be explained by the general steric hindrance in the bulky PEGylated form. Mutations at the L5A, position in apelin-13 (corresponding to L28A in apelin-36) had modest effect on apelin receptor binding and signalling in cultured cells stably expressing the receptor [28,29]. Our data for the apelin-36 analogues in experiments using native rat and human receptor confirm that the mutation at this position in the longer apelin isoform does not adversely affect binding affinity for the apelin receptor.

In our cell based assays, we confirmed the decreased β-arrestin activation initially reported by Galon-Tilleman et al. [20], who found that apelin-36-[L28A] and apelin-36-[L28C(30kDa-PEG)] were 100 and 10,000-fold respectively less potent compared to the endogenous apelin-36, although in our study the reduction in potency of apelin-36-[L28C(30kDa-PEG)] was only 1400-fold. We have now determined the potency of these analogues and found them to be less effective than apelin-36 in both the G protein-dependent cAMP accumulation and β-arrestin assays but this potency reduction was more apparent in the β-arrestin assay indicating a degree of G protein bias for these analogues compared to apelin-36. Further analysis confirmed both were G protein biased agonists with bias factors of 13 and 58, respectively. In addition, alanine substitutions of proline and leucine at positions 13 and 28, apelin-36-[A13 A28], resulted in approximately 10-fold decrease in potency in the β-arrestin assay compared to cAMP assay. The bias factor for this peptide was 17, suggesting that alanine substitution at these positions promote G protein signalling over β-arrestin recruitment. Hence, our findings are consistent with enhanced functional selectivity (bias) towards G protein-dependent signalling by these apelin-36 analogues. We have previously reported that the apelin receptor is tractable to development of biased agonists and have identified a biased apelin peptide, MM07 [25,30], generated by N-terminal cyclisation with flanking cysteine residues as well as the first small molecule biased apelin receptor agonist, CMF-019 [22].

Using molecular dynamic simulation and site-directed mutagenesis it was reported that upon binding to the receptor the C-terminal phenylalanine residue of apelin peptides is embedded by Trp^152^ in the transmembrane domain IV and by Trp^259^ and Phe^255^ in transmembrane domain VI of the apelin receptor. Deletion of this amino acid abrogated β-arrestin-mediated apelin receptor internalisation but did not affect cAMP inhibition [31]. Moreover, we and others have also shown that deletion of this phenylalanine residue does not affect binding and that the resulting peptide is still functional at the receptor [32,33]. Consistent with this, alanine substitution of the apelin-36 C-terminal phenylalanine, apelin-36[F13A], biased responses towards G protein dependent signalling with a bias factor of 28.

Apelin receptor-mediated metabolic effects have been well characterised. For example, apelin inhibited isoproterenol-induced free fatty acid release in isolated adipocytes [34]. The apelin effect was attenuated by inhibition of Gα_q_ with Gp2A or Gα_i_ with pertussis toxin or AMP-activated protein kinase (AMPK) by either compound C or dorsomorphin [18,34]. Apelin also stimulates glucose transport in skeletal muscle and adipose tissue via the activation of AMPK and eNOS demonstrated using mice expressing a muscle-specific dominant-negative AMPK mutant [17,18,35,36]. Recently, in a diet-induced obesity mouse model it was shown that acute administration of apelin-13 and analogues with enhanced in vitro plasma stability resulted in improved glucose homeostasis and insulin secretion [37,38]. Similarly in the same model chronic administration of [Pyr^1^]apelin-13 systemically over 28 days, exhibited anti-diabetic properties by directly targeting lipid metabolism thus reducing hepatic steatosis [39]. This was likely as a result of improved insulin sensitivity and glucose uptake by muscle rather than a direct effect on hepatocytes.

There is still an unmet need for the development of therapeutics that could improve the management, and potentially treat type 2 diabetes mellitus and its associated metabolic syndromes. This is particularly important since over 415 million people globally suffer from type 2 diabetes mellitus and the figure is expected to rise to 642 million by 2040 [40]. The apelin receptor signalling pathway has been shown to modulate several physiological and pathological states. It has important beneficial effects in cardiovascular and metabolic diseases including myocardial infarction [15,41,42], hypertension [24,30,43], obesity and type 2 diabetes [44,45]. This suggests that apelin-based therapeutics may present a novel mechanism for clinical management or treatment of metabolic syndrome. However, like most hormonal peptides their activity is often short-lived owing to rapid degradation by endopeptidases. This has been the impetus to develop apelin peptides with improved pharmacokinetic properties. Our study therefore reinforces this by demonstrating that the beneficial effects of these analogues in diet-induced obesity mice model were mediated via the apelin receptor, making it a novel therapeutic target in diabetes.

## 5 Conclusions

**Fig. 5:**
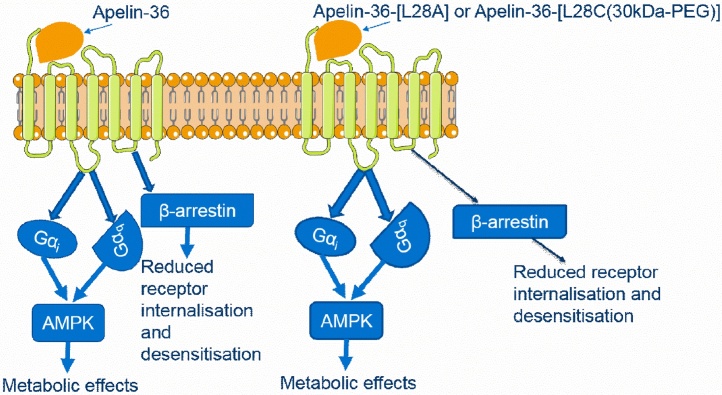
**Proposed signalling cascade activated by apelin-36-[L28A] and apelin-36-[L28C(30kDa-PEG)] upon binding to the apelin receptor**. On the left, apelin-36 recruits Gα_i_ and Gα_q_ signalling following receptor binding resulting in the activation of AMP-dependent kinases. However, it will also activate β-arrestin recruitment, which will result in receptor desensitisation and degradation or recycling back to the cell surface. On the other hand, apelin-36-[L28A] and apelin-36-[L28C(30kDa-PEG)] on the right recruits Gα_i_ and Gα_q_ signalling resulting in the activation of AMP-dependent kinase group of enzymes which mediates the downstream beneficial metabolic effects. There is reduced receptor internalization and desensitization, which is likely to prolong the agonists activity of the two peptides compared to apelin-36.

Although initially reported to mediate their effects in an apelin receptor-independent manner we have now shown that apelin-36-[L28A] and apelin-36-[L28C(30kDa-PEG)] bind to the apelin receptor with nanomolar affinities. Our data provide evidence that these peptides are G protein biased apelin receptor agonists and this pharmacological profile is consistent with their reported beneficial *in vivo* metabolic actions via Gα_i_ and/or Gα_q_ signalling pathways (Fig. 5).

## Grant Information

We thank the following for support: Wellcome Trust [<GN1>WT107715/Z/15/Z to APD, JJM], W</GN1>ellcome Trust Programme in Metabolic and Cardiovascular Disease [203814/Z/16/Ato TLW]; Astra-Zeneca, Cambridge Biomedical Research Centre Biomedical Resources Grant, University of Cambridge, Cardiovascular Theme, <GN3>RG64226.</GN3>

## Author contribution

DN designed and performed experiments, analysed data, wrote manuscript, REK and TLW performed experiments, MB, PA, LJ provided resources and funding, JJM, APD provided experimental design, data analysis, wrote manuscript, supervision of project and funding.

MB, PA and LS are employees of AstraZeneca (UK) Plc.

## Declaration of Competing Interest

MB, PA and LS are employees of AstraZeneca (UK) Plc.

## References

[1] O’Dowd B.F., Heiber M., Chan A., Heng H.H., Tsui L.C., Kennedy J.L., Shi X., Petronis A., George S.R., Nguyen T. (1993). A human gene that shows identity with the gene encoding the angiotensin receptor is located on chromosome 11. Gene.

[2] Tatemoto K., Hosoya M., Habata Y., Fujii R., Kakegawa T., Zou M.X., Kawamata Y., Fukusumi S., Hinuma S., Kitada C., Kurokawa T., Onda H., Fujino M. (1998). Isolation and characterization of a novel endogenous peptide ligand for the human APJ receptor. Biochem. Biophys. Res. Commun..

[3] Pitkin S.L., Maguire J.J., Bonner T.I., Davenport A.P. (2010). International Union of Basic and Clinical Pharmacology. LXXIV. Apelin receptor nomenclature, distribution, pharmacology, and function. Pharmacol. Rev..

[4] De Mota N., Reaux-Le Goazigo A., El Messari S., Chartrel N., Roesch D., Dujardin C., Kordon C., Vaudry H., Moos F., Llorens-Cortes C. (2004). Apelin, a potent diuretic neuropeptide counteracting vasopressin actions through inhibition of vasopressin neuron activity and vasopressin release. Proc. Natl. Acad. Sci. U. S. A..

[5] Maguire J.J., Kleinz M.J., Pitkin S.L., Davenport A.P. (2009). [Pyr1]apelin-13 identified as the predominant apelin isoform in the human heart: vasoactive mechanisms and inotropic action in disease. Hypertension.

[6] Zhen E.Y., Higgs R.E., Gutierrez J.A. (2013). Pyroglutamyl apelin-13 identified as the major apelin isoform in human plasma. Anal. Biochem..

[7] Kleinz M.J., Davenport A.P. (2004). Immunocytochemical localization of the endogenous vasoactive peptide apelin to human vascular and endocardial endothelial cells. Regul. Pept..

[8] Kleinz M.J., Davenport A.P. (2005). Emerging roles of apelin in biology and medicine. Pharmacol. Ther..

[9] O’Carroll A.M., Lolait S.J., Harris L.E., Pope G.R. (2013). The apelin receptor APJ: journey from an orphan to a multifaceted regulator of homeostasis. J. Endocrinol..

[10] Yang P., Maguire J.J., Davenport A.P. (2015). Apelin, Elabela/Toddler, and biased agonists as novel therapeutic agents in the cardiovascular system. Trends Pharmacol. Sci..

[11] Read C., Nyimanu D., Williams T.L., Huggins D.J., Sulentic P., Macrae R.G.C., Yang P., Glen R.C., Maguire J.J., Anthony P. (2019). International Union of Basic and Clinical Pharmacology XXX. Structure and pharmacology of the apelin receptor with a recommendation that Elabela/Toddler is a second endogenous peptide ligand. Pharmacol. Rev..

[12] El Messari S., Iturrioz X., Fassot C., De Mota N., Roesch D., Llorens-Cortes C. (2004). Functional dissociation of apelin receptor signaling and endocytosis: implications for the effects of apelin on arterial blood pressure. J. Neurochem..

[13] Japp A.G., Cruden N.L., Amer D.A.B., Li V.K.Y., Goudie E.B., Johnston N.R., Sharma S., Neilson I., Webb D.J., Megson I.L., Flapan A.D., Newby D.E. (2008). Vascular effects of apelin in vivo in man. J. Am. Coll. Cardiol..

[14] Chen M.M., Ashley E.A., Deng D.X.F., Tsalenko A., Deng A., Tabibiazar R., Ben-Dor A., Fenster B., Yang E., King J.Y., Fowler M., Robbins R., Johnson F.L., Bruhn L., McDonagh T., Dargie H., Yakhini Z., Tsao P.S., Quertermous T. (2003). Novel role for the potent endogenous inotrope apelin in human cardiac dysfunction. Circulation.

[15] Berry M.F., Pirolli T.J., Jayasankar V., Burdick J., Morine K.J., Gardner T.J., Woo Y.J. (2004). Apelin has in vivo inotropic effects on normal and failing hearts. Circulation.

[16] Tero-Pekka Alastalo D.P., Li M., de Jesus Perez V., Hirofumi Sawada L.W., Wang J.K., Koskenvuo M., Bruce M.R., Freeman A., Chang H.Y. (2011). Disruption of PPARγ/β-catenin–mediated regulation of apelin impairs BMP-induced mouse and human pulmonary arterial EC survival. J. Clin. Invest..

[17] Dray C., Knauf C., Daviaud D., Waget A., Boucher J., Buléon M., Cani P.D., Attané C., Guigné C., Carpéné C., Burcelin R., Castan-Laurell I., Valet P. (2008). Apelin stimulates glucose utilization in normal and obese insulin-resistant mice. Cell Metab..

[18] Yue P., Jin H., Aillaud M., Deng A.C., Azuma J., Asagami T., Kundu R.K., Reaven G.M., Quertermous T., Tsao P.S. (2010). Apelin is necessary for the maintenance of insulin sensitivity. Am. J. Physiol. Endocrinol. Metab..

[19] Higuchi K., Masaki T., Gotoh K., Chiba S., Katsuragi I., Tanaka K., Kakuma T., Yoshimatsu H. (2007). Apelin, an APJ receptor ligand, regulates body adiposity and favors the messenger ribonucleic acid expression of uncoupling proteins in mice. Endocrinology.

[20] Galon-Tilleman H., Yang H., Bednarek M.A., Spurlock S.M., Paavola K.J., Ko B., To C., Luo J., Tian H., Jermutus L., Grimsby J., Rondinone C.M., Konkar A., Kaplan D.D. (2017). Apelin-36 modulates blood glucose and body weight independently of canonical APJ receptor signaling. J. Biol. Chem..

[21] Parrott M.C., DeSimone J.M. (2012). Relieving PEGylation. Nat. Chem..

[22] Read C., Fitzpatrick C.M., Yang P., Kuc R.E., Maguire J.J., Glen R.C., Foster R.E., Davenport A.P. (2016). Cardiac action of the first G protein biased small molecule apelin agonist. Biochem. Pharmacol..

[23] McPherson G.A. (1985). Analysis of radioligand binding experiments: a collection of computer programs for the IBM PC. J. Pharmacol. Methods.

[24] Yang P., Read C., Kuc R.E., Buonincontri G., Southwood M., Torella R., Upton P.D., Crosby A., Sawiak S.J., Carpenter T.A., Glen R.C., Morrell N.W., Maguire J.J., Davenport A.P. (2017). Elabela/Toddler is an endogenous agonist of the apelin APJ receptor in the adult cardiovascular system, and exogenous administration of the peptide compensates for the downregulation of its expression in pulmonary arterial hypertension. Circulation.

[25] Yang P., Read C., Kuc R.E., Nyimanu D., Williams T.L., Crosby A., Buonincontri G., Southwood M., Sawiak S.J., Morrell N.W., Davenport A.P., Maguire J.J. (2019). A novel cyclic biased agonist of the apelin receptor, MM07, is disease modifying in the rat monocrotaline model of pulmonary arterial hypertension. Br. J. Pharmacol..

[26] van der Westhuizen E.T., Breton B., Christopoulos A., Bouvier M. (2014). Quantification of ligand bias for clinically relevant β2-adrenergic receptor ligands: implications for drug taxonomy. Mol. Pharmacol..

[27] Kenakin T., Watson C., Muniz-Medina V., Christopoulos A., Novick S. (2012). A simple method for quantifying functional selectivity and agonist bias. ACS Chem. Neurosci..

[28] Medhurst A.D., Jennings C.A., Robbins M.J., Davis R.P., Ellis C., Winborn K.Y., Lawrie K.W.M., Hervieu G., Riley G., Bolaky J.E., Herrity N.C., Murdock P., Darker J.G. (2003). Pharmacological and immunohistochemical characterization of the APJ receptor and its endogenous ligand apelin. J. Neurochem..

[29] Fan X., Zhou N., Zhang X., Mukhtar M., Lu Z., Fang J., DuBois G.C., Pomerantz R.J. (2003). Structural and Functional Study of the Apelin-13 Peptide, an Endogenous Ligand of the HIV-1 Coreceptor, APJ. Biochemistry.

[30] Brame A.L., Maguire J.J., Yang P., Dyson A., Torella R., Cheriyan J., Singer M., Glen R.C., Wilkinson I.B., Davenport A.P. (2015). Design, characterization, and first-in-human study of the vascular actions of a novel biased apelin receptor agonist. Hypertension.

[31] Iturrioz X., Gerbier R., Leroux V., Alvear-Perez R., Maigret B., Llorens-Cortes C. (2010). By interacting with the C-terminal phe of apelin, Phe255 and Trp259 in Helix vi of the apelin receptor are critical for internalization. J. Biol. Chem..

[32] Wang W., McKinnie S.M.K., Farhan M., Paul M., McDonald T., McLean B., Llorens-Cortes C., Hazra S., Murray A.G., Vederas J.C., Oudit G.Y. (2016). Angiotensin-converting enzyme 2 metabolizes and partially inactivates Pyr-Apelin-13 and Apelin-17: physiological effects in the cardiovascular system. Hypertension.

[33] Yang P., Kuc R.E., Brame A.L., Dyson A., Singer M., Glen R.C., Cheriyan J., Wilkinson I.B., Davenport A.P., Maguire J.J. (2017). [Pyr1]Apelin-13(1–12) is a biologically active ACE2 metabolite of the endogenous cardiovascular peptide [Pyr1]Apelin-13. Front. Neurosci..

[34] Yue P., Jin H., Xu S., Aillaud M., Deng A.C., Azuma J., Kundu R.K., Reaven G.M., Quertermous T., Tsao P.S. (2011). Apelin decreases lipolysis via g_q_, g_i_, and AMPK-Dependent mechanisms. Endocrinology.

[35] Dray C., Sakar Y., Vinel C., Daviaud D., Masri B., Garrigues L., Wanecq E., Galvani S., Negre–Salvayre A., Barak L.S., Monsarrat B., Burlet–Schiltz O., Valet P., Castan–Laurell I., Ducroc R. (2013). The intestinal glucose–Apelin cycle controls carbohydrate absorption in mice. Gastroenterology.

[36] Attane C., Daviaud D., Dray C., Dusaulcy R., Masseboeuf M., Prevot D., Carpene C., Castan-Laurell I., Valet P. (2011). Apelin stimulates glucose uptake but not lipolysis in human adipose tissue ex vivo. J. Mol. Endocrinol..

[37] O’Harte F.P.M., Parthsarathy V., Hogg C., Flatt P.R. (2017). Acylated apelin-13 amide analogues exhibit enzyme resistance and prolonged insulin releasing, glucose lowering and anorexic properties. Biochem. Pharmacol..

[38] O’Harte F.P.M., Parthsarathy V., Hogg C., Flatt P.R. (2018). Apelin-13 analogues show potent in vitro and in vivo insulinotropic and glucose lowering actions. Peptides.

[39] Bertrand C., Pradère J.-P., Geoffre N., Deleruyelle S., Masri B., Personnaz J., Le Gonidec S., Batut A., Louche K., Moro C., Valet P., Castan-Laurell I. (2018). Chronic apelin treatment improves hepatic lipid metabolism in obese and insulin-resistant mice by an indirect mechanism. Endocrine.

[40] Zimmet P., Alberti K.G., Magliano D.J., Bennett P.H. (2016). Diabetes mellitus statistics on prevalence and mortality: facts and fallacies. Nat. Rev. Endocrinol..

[41] Japp A.G., Newby D.E. (2008). The apelin-APJ system in heart failure. Pathophysiologic relevance and therapeutic potential. Biochem. Pharmacol..

[42] Simpkin J.C., Yellon D.M., Davidson S.M., Lim S.Y., Wynne A.M., Smith C.C.T. (2007). Apelin-13 and apelin-36 exhibit direct cardioprotective activity against ischemiareperfusion injury. Basic Res. Cardiol..

[43] Przewlocka-Kosmala M., Kotwica T., Mysiak A., Kosmala W. (2011). Reduced circulating apelin in essential hypertension and its association with cardiac dysfunction. J. Hypertens..

[44] Castan-Laurell I., Dray C., Attané C., Duparc T., Knauf C., Valet P. (2011). Apelin, diabetes, and obesity. Endocrine.

[45] Bertrand C., Valet P., Castan-Laurell I. (2015). Apelin and energy metabolism. Front. Physiol..

